# Supplementary material for: Desaturase-dependent secretory functions of hepatocyte-like cells control systemic lipid metabolism during starvation in Drosophila
Source: Nat Commun. 2025 Nov 21;16:10409. doi: 10.1038/s41467-025-66571-5 (PMC12644726; doi:10.1038/s41467-025-66571-5)
Supplement: Supplementary file 1 — Supplementary Information [file 41467_2025_66571_MOESM1_ESM.pdf]

Supplementary Figures:

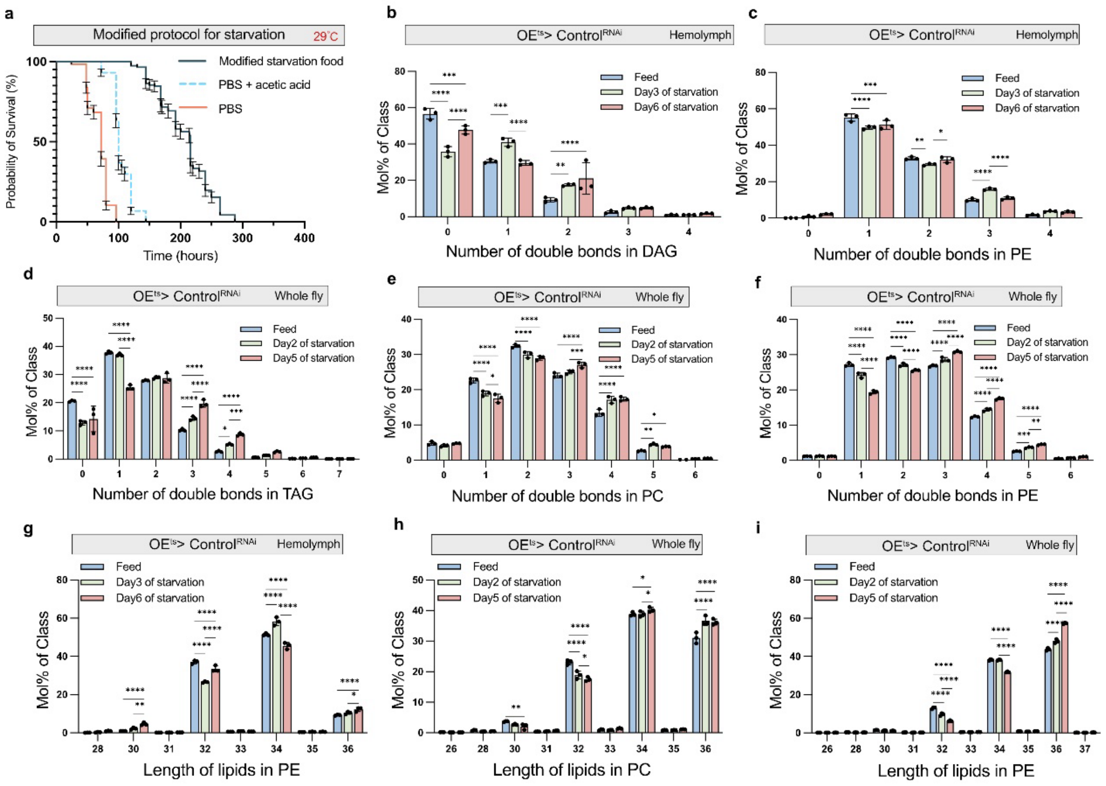

Supplementary Figure 1: Lipidomic analysis of hemolymph or whole fly sample at different days of starvation

a Comparison of modified starvation protocol (incl. acetic acid supplementation) and normal PBS starvation protocol plus acetic acid supplementation. N=120. **b-f** The number of double bonds in different major lipid classes in hemolymph or whole samples. DAG, diglyceride; TAG, triglyceride; PE, phosphatidyl ethanolamine; PC, phosphatidylcholine. n=3, statistical tests: two-way ANOVA with Tukey's multiple comparisons test. **g-i** Length of lipids in different lipid classes, n=3, statistical tests: two-way ANOVA with Tukey's multiple comparisons test. *, P< 0.05; **, P<0.01; ***, P<0.001; ****, P<0.0001. Source data for plots and exact p-value are provided as a source data file.

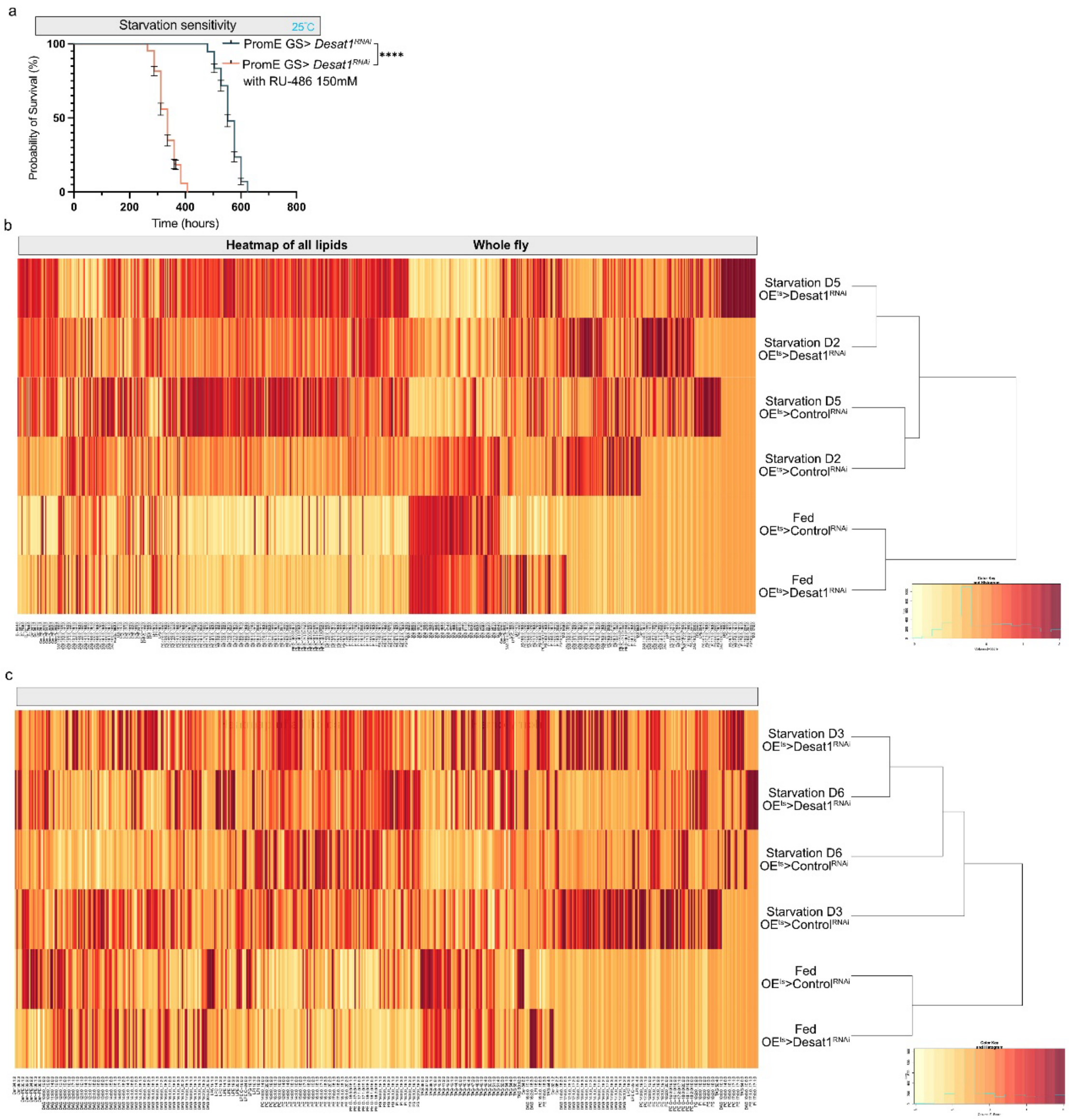

Supplementary Figure 2: Starvation sensitivity and lipidomics heatmap

a Starvation assay performed at 25°C by using PromE GS> Desat1^{RNAi} flies with or without RU486, N=150, ****: P<0.0001. **b** Heatmap from whole-fly and hemolymph lipidomic profiles in starvation and fed conditions, n=3. Source data for plots and exact p-value are provided as a source data file.

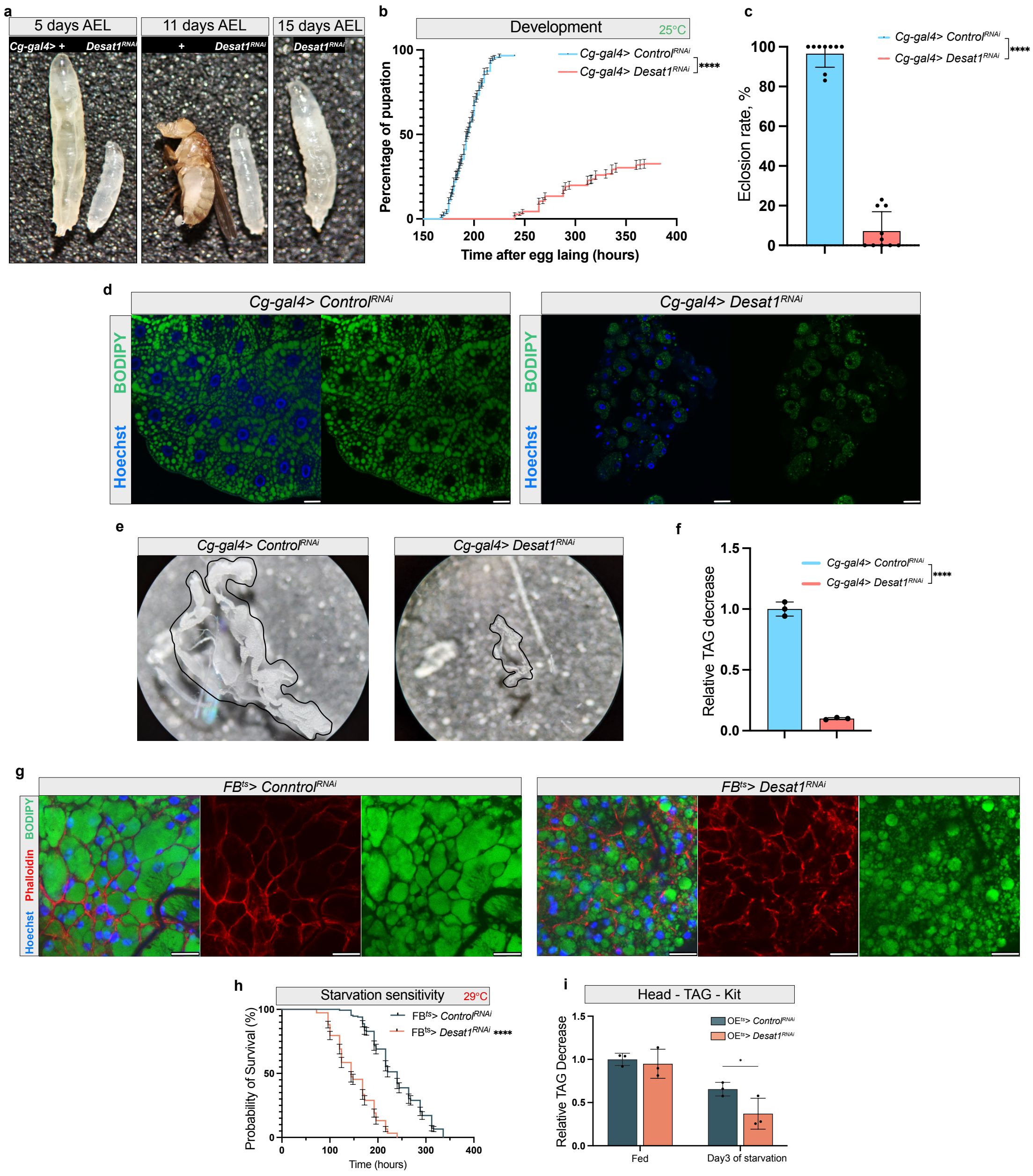

Supplementary Figure 3: Role of Desat1 in the fat body

a Representative image of flies at different development stages. Flies with fat body (FB)-specific *Desat1* KD showed a strong development delay, n>4. **b, c** Percentage of pupation and eclosion of control or FB *Desat1* KD group, n=9. **d** Representative images of FB LDs stained by BODIPY in control or *Desat1* KD group, n=3, scale bars: 20 μm. **e** Representative images of FB morphology in control or FB *Desat1* KD group. Black lines mark the FB. **f** TAG level of whole larva measured by TAG kit, N=3. Statistical tests: unpaired t test. AEL, after egg laying, scale bars: 20 μm. **g** Representative image of adult flies with fat body *Desat1* KD, FB^{ts}: +; Dcg-gal4; tub-gal80ts, n=3, scale bars: 20 μm. **h** Starvation sensitivity of flies with fat body-specific *Desat1* KD in adult stage at 29℃, n=150. **i** TAG level of head from adult with *Desat1* KD measured by TAG kit, N=3. *, P< 0.05; **, P<0.01; ***, P<0.001; ****, P<0.0001. Source data for plots and exact p-value are provided as a source data file.

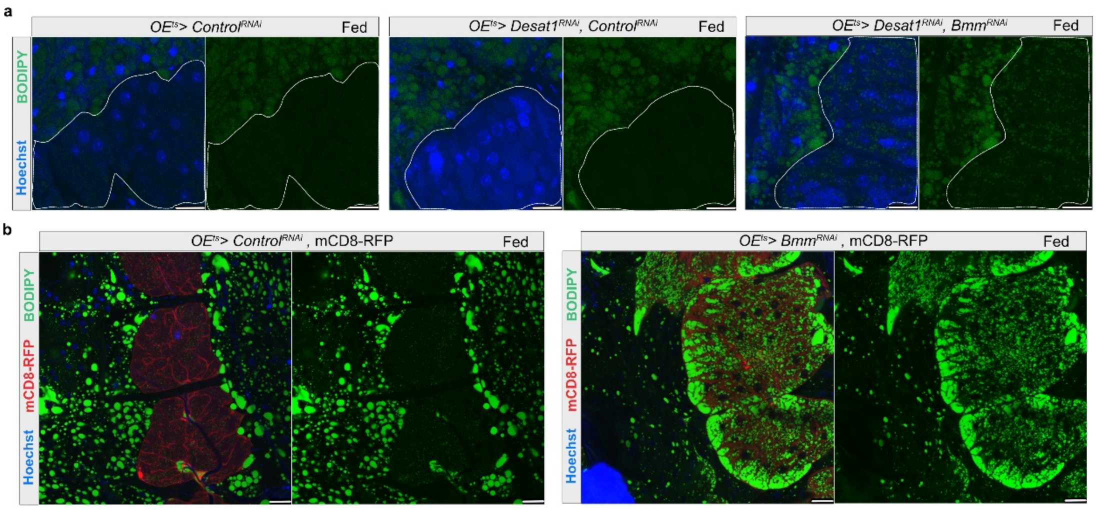

Supplementary Figure 4: Role of bmm in oenocytes

a, b, Representative images of LDs stained by BODIPY in control, *bmm* KD or *Desat1*/*bmm* KD groups. n=3, mCD8-FRP expression marks oenocytes (white dashed lines), scale bars: 20 μm.

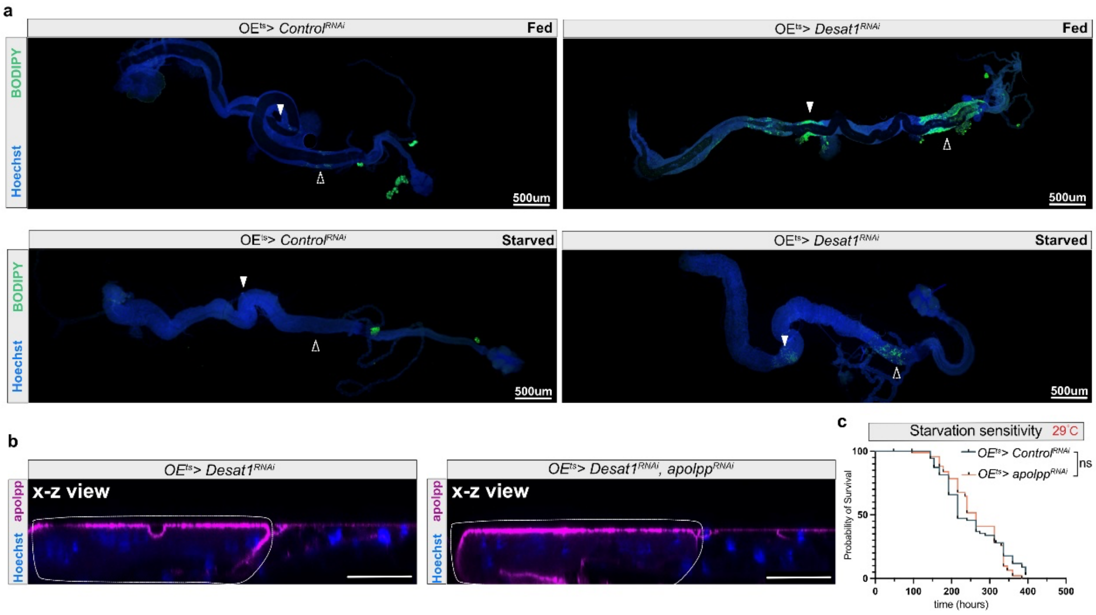

Supplementary Figure 5: Lpp accumulation in oenocytes is not cell-autonomous and affects gut lipid metabolism

a Representative image of LDs visualized by BODIPY in adult gut. In both fed and starved condition, flies with oenocyte-specific *Desat1* KD exhibit a LDs accumulation compared with control group. n=3, scale bars: 500 µm. **b** Representative x-z side view images of apolpp in *Desat1* KD and *Desat1*/*apolpp* KD group with dashed white lines marking the oenocytes, scale bars: 20 µm. **c** Starvation sensitivity assay between control and *apolpp* KD groups at 29℃. N=80, P value was calculated using Log-rank (Mantel-Cox) test. Note that expression of the same *apolpp* RNAi in FB led to developmental arrest (our own data and Ref.34), demonstrating functionality of the RNAi. Source data for plots and exact p-value are provided as a source data file.

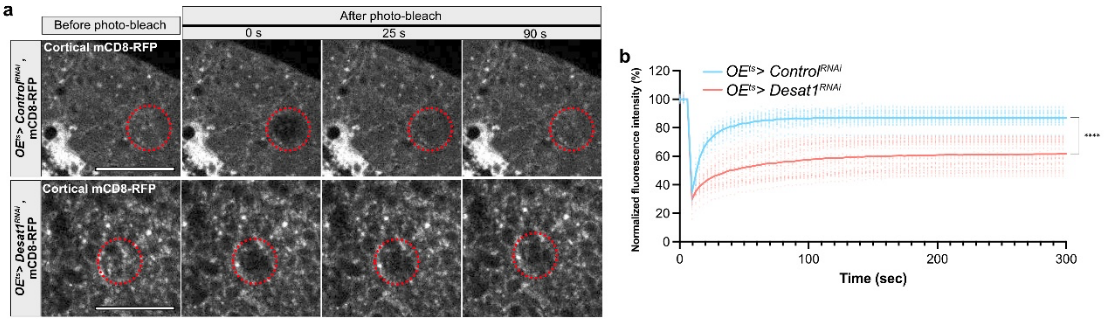

Supplementary Figure 6: Diffusion of mCD8-RFP on oenocyte surface is restricted upon *Desat1* KD

a Fluorescence recovery after photobleaching (FRAP) of surface mCD8-RFP shows impaired diffusion in oenocytes with *Desat1* KD compared to controls. Red dashed lines show the region of photo-bleaching on the oenocyte surface, scale bar: 10 μm. **b** The intensity of fluorescence is plotted over time. The dip at 10 seconds reflects the photobleaching event. ****P<0.0001, *OE*^{ts}> *Desat1*^{RNAi} vs. *OE*^{ts}> *Control*^{RNAi}, n=35, each data point represented a FRAP experiment performed in a cell surface. Statistical tests: two-way ANOVA. Source data for plots and exact p-value are provided as a source data file.

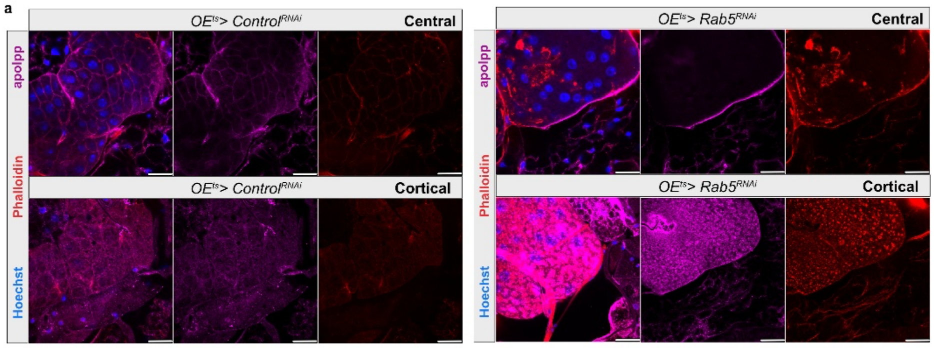

Supplementary Figure 7: *Rab5* silencing leads to apolpp and actin accumulation in oenocytes

a Representative central and cortical sections of apolpp and phalloidin in control and *Rab5* KD groups, n=3, scale bars: 20 µm.

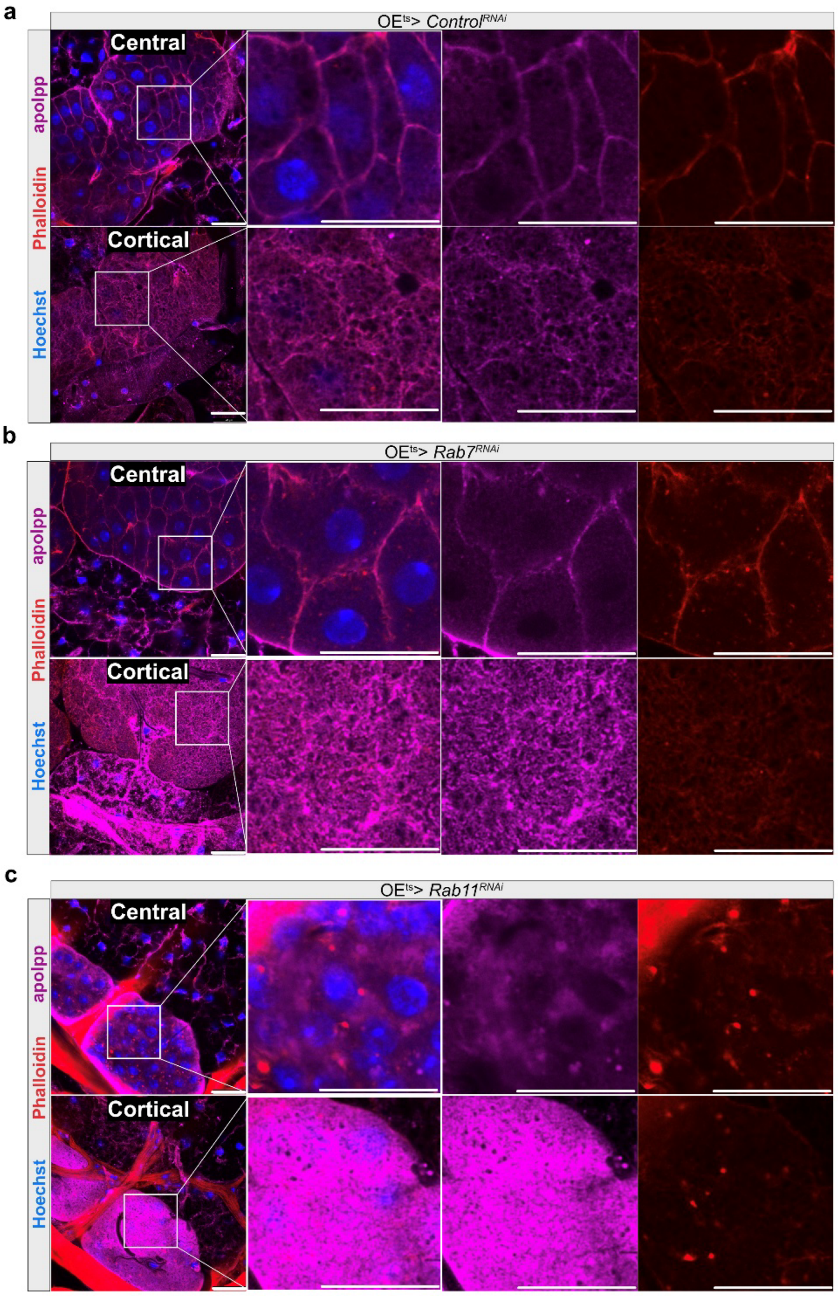

Supplementary Figure 8: *Rab11,* but not Rab7, silencing leads to mild apolpp accumulation in oenocytes

a-c Representative central and cortical sections of apolpp and phalloidin in *Control*^{RNAi}, *Rab7*^{RNAi} or *Rab11*^{RNAi} groups, n=3, scale bars: 20 μm.

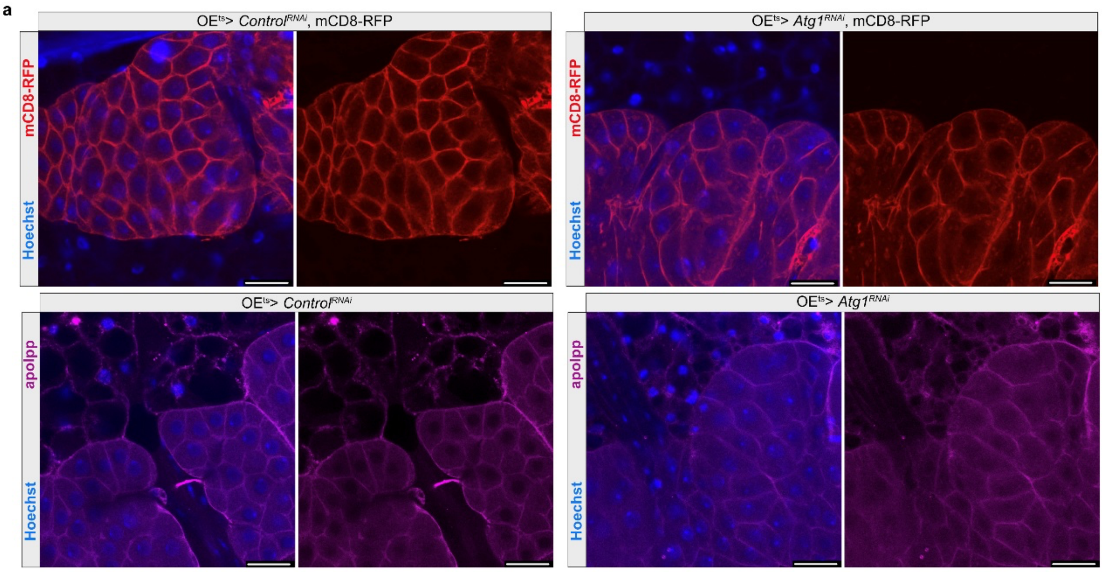

Supplementary Figure 9: Atg1 silencing does not cause apolpp accumulation in oenocytes.

a Representative image of apolpp and mCD8-RFP in control and Atg1 KD group. n=2, scale bars: 20 µm.

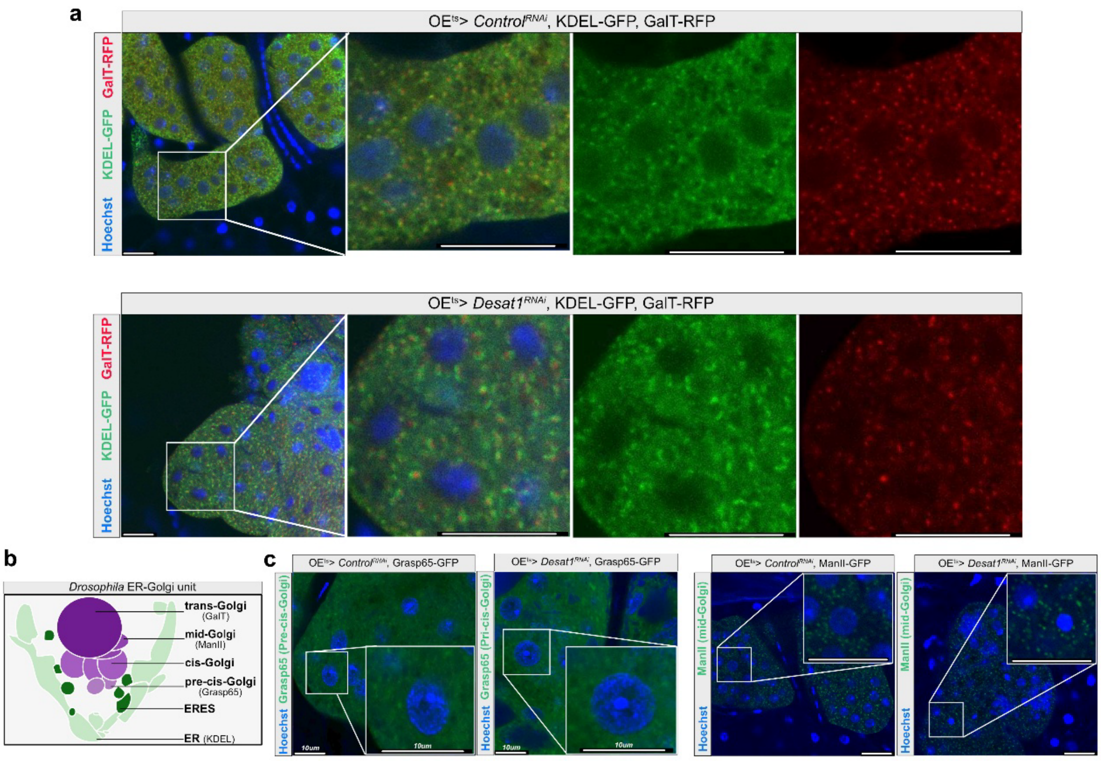

Supplementary Figure 10: ER-Golgi unit of oenocytes with or without *Desat1* KD

a Representative image of ER and trans-Golgi from oenocytes with or without *Desat1* KD, visualized by KEDL-GFP or GalT-RFP. scale bars: 20μm. **b** the schematic diagram of ER-Golgi unit in oenocytes. **c** Representative image of pre-cis-Golgi or mid-Golgi from oenocytes, visualized by Grasp65-GFP or ManII-GFP respectively. scale bars: 10μm (Grasp65-GFP), 20μm (ManII-GFP), N=3.

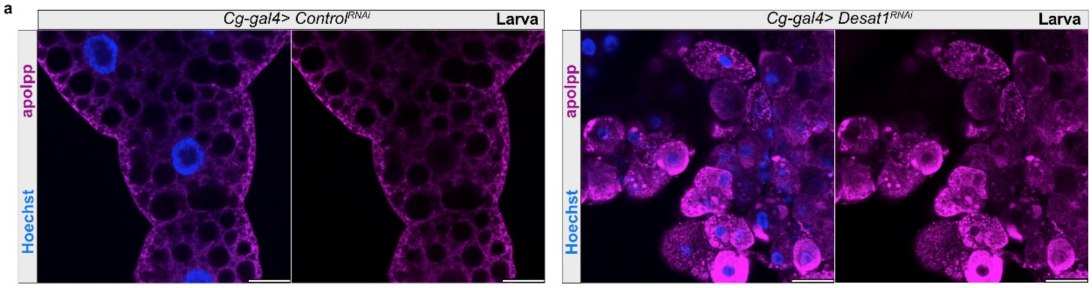

Supplementary Figure 11: Apolpp secretion in fat body with Desat1 deficiency

a Representative fat body images of apolpp in larva, n=2, scale bars: 20μm.

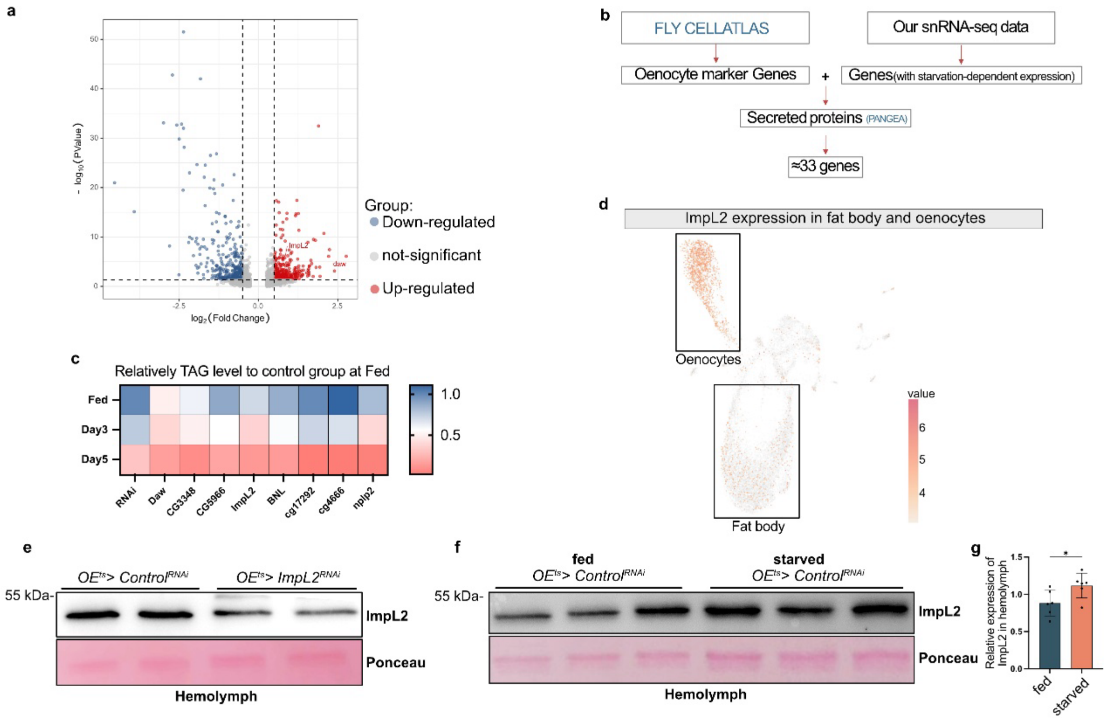

Supplementary Figure 12: ImpL2 secretion by oenocytes is increased in starvation

a Differentially expressed genes in oenocytes (day 2 of starvation vs. fed condition) were visualized using a volcano map (Fold change >0.5 and P value < 0.05). **b** A protocol of gene selection. **c** Relative TAG level measured by kit from whole flies with different candidate gene KD specifically in oenocytes, n=4. **d** *ImpL2* expression pattern from DRSC RNA-seq explorer showed a high expression level of *Impl2* in oenocytes compared with fat body. **e** Western blot analysis of ImpL2 level in hemolymph from control or *OE^{ts}>$ImpL2^{RNAi}$* flies, n=2. **f** Western blot analysis of ImpL2 at fed or starvation condition in hemolymph. **g** Quantification of ImpL2 level in hemolymph in fed or starvation condition, n=3, statistical tests: unpaired t test, *p<0.05. Source data for plots and exact p-value are provided as a source data file.

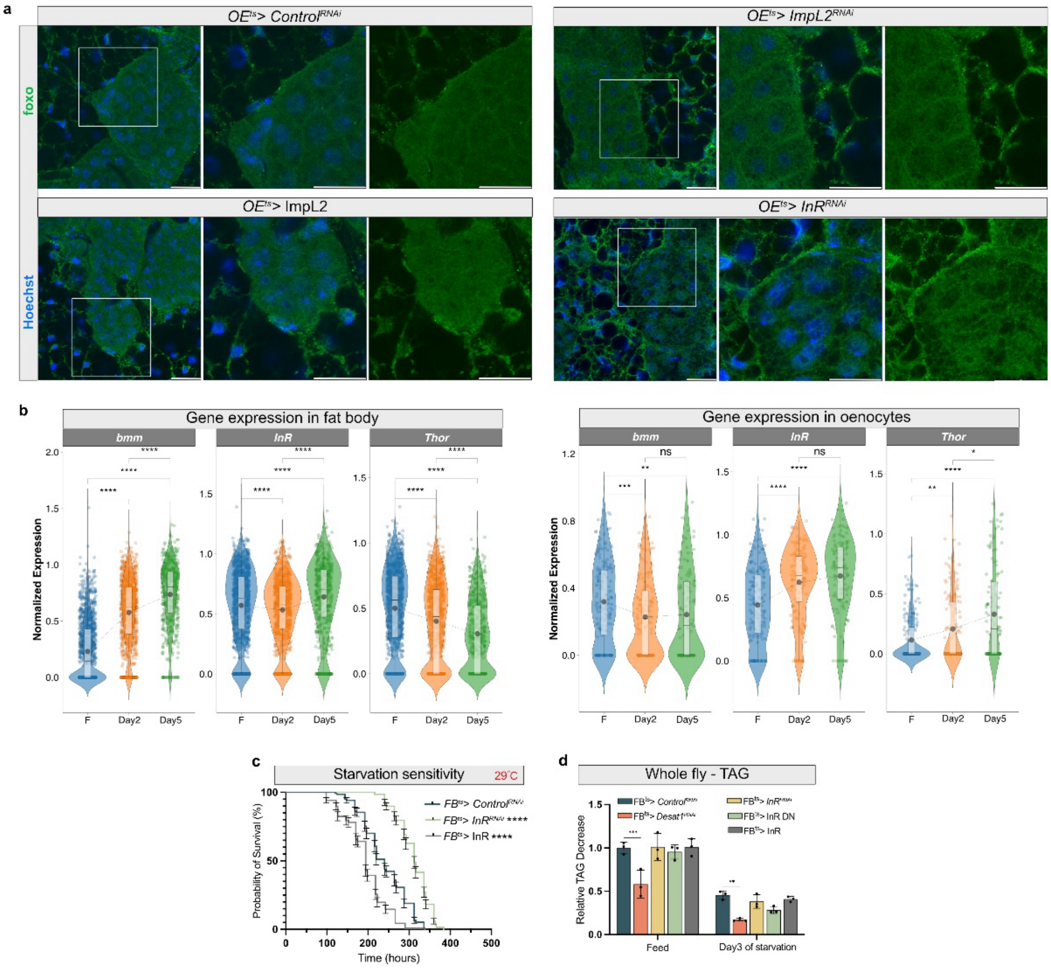

Supplementary Figure 13: IIS in oenocytes and FB

a Representative images of foxo in adult oenocytes and FB upon oenocyte-specific ImpL2 OE, ImpL2 KD and InR KD. scale bars: 20μm. **b** mRNA level of foxo target genes (bmm, InR and Thor) in fed (F) conditions and on day 2 and 5 of our starvation protocol in FB or oenocytes, respectively, as measured by sn-RNA-seq. P value was calculated using Wilcoxon test. **c** Starvation sensitivity of flies with FB-specific InR KD or overexpression. n=140. P value was calculated using Log-rank (Mantel-Cox) test. ****, P<0.0001. **d** TAG level of flies with FB-specific InR KD, InR DN (DN, dominant negative, InR K1409A) and InR overexpression. Desat1 KD was used as a positive control. n=3. Statistical tests: two-way ANOVA. **, P<0.01; ***, P<0.001; ****, P<0.0001. Source data for plots and exact p-value are provided as a source data file.

Supplementary Tables:

Name	Reference
W [1118]	VDRC 60000
w; P{UAS-Desat1-RNAi-GD2950}v33338	VDRC 33338
w; ; P{UAS-desat1-RNAi-KK107747}v104350	VDRC 104350
P{w[+mC]=Desat1-GAL4.E800}2M, P{w[+mC]=tubP-GAL80[ts]}20	Bloo. #65406
P{w[+mC]=Desat1-GAL4.E800}4M, P{w[+mC]=tubP-GAL80[ts]}2	Bloo. #65407
w; Desat1 RNAi; PromE gal80^{ts}	This work
w; mCD8-RFP; PromE gal80^{ts}	This work
PromE gal80^{ts}, CyO-GFP; Apolpp-GFP, TM6B, Tb	This work
P[mw, UAS-mCD8-RFP]/(CyO)	From Yohanns Bellaiche
y, w; P[mw, UAS-mCD8-GFP]	Bloo. #5137
y[1], w[*]; UAS-Albumin-mCherry	This work
w;; Lpp-gal4	From Bruno Lemaitre
y[1] v[1]; P{y[+t7.7] v[+t1.8]=TRiP.UAS-apolppp RNAi}attP2	Bloo. #28946
y[1] v[1]; P{y[+t7.7] v[+t1.8]=TRiP UAS-LIMK1-RNAi}attP40	Bloo. #42576
y[1] sc[*] v[1] sev[21]; P{y[+t7.7] v[+t1.8]=TRiP UAS-tsr-RNAi}attP2	Bloo. #65055
y[1] w[*]; P{w[+mC]=UAS-tsr.N}2.2.1/TM6B, P{y[+t7.7] ry[+t7.2]=Car20y}TPN1, Tb[1]	Bloo. #9235
w; P[mw, UAS-Rab5-RNAi]KK103945	VDRC v103945
FlyFos027263(pRedFlp-Hgr)(Rfabg30509::2XTY1-SGFP-V5-preTEV-BLRP-	VDRC

3XFLAG)dFRT	v318255
;; UAS-ImpL2-HA OE	FlyORF (F001712)
w; P[mw, UAS-daw-RNAi]	VDRC v5085
w; P[mw, UAS-ImpL2-RNAi]	VDRC 30930
W; P[mw, UAS-daw-RNAi]; PromE gal80ts	This work
w[*]; P{w[+mC]=UAS-GFP.KDEL}11.1; P[mw, UASp-RFP.Golgi]10	This work
w; P[mw, UAS-Rab7-RNAi]GD40338	VDRC v40338
w; P[mw, UAS-Rab11-RNAi]GD22198	VDRC v22198
w[*]; UAS-Grasp65-GFP	Bloo. #8507
;Dcg-gal4;tub-gal80ts,UAS-GFP	From W. Mike Henne
W; P{UAS-ATG1-RNAi-GD7149}	VDRC v16133
w[1118]; UAS-ManII-EGFP	Bloo. #65248
y[1] w[1118]; P{y[+t7.7] w[+mC]=r5-BPnlsLexA::GADflUw}attP40/CyO	From Seung K. Kim
w;;LexAop-Bmm^{RNAi}	This work
y[1] v[1];; P{y[+t7.7] v[+t1.8]=TRiP.JF01482}attP2 (UAS-InR-RNAi)	Bloo. #31037
y[1] w[1118]; P{w[+mC]=UAS-InR.R418P}2	Bloo. # 8250
y[1] w[1118]; P{w[+mC]=UAS-InR.K1409A}3	Bloo. # 8253
w; P[mw, Cg-Gal4];+	From Samuel Liegeois
w;; bmm[1]/TM3, Sb (floating)	From Ronald Kuehnlein
w; P{GD5139}v37877 UAS-brummer-dsRNA	VDRC #37877

Supplementary Table 1: All flies strains used in this study.

Gene	Forward	Reverse
RPL32	TCTGTTGTCGATACCCTTGGGCTT	AAGAAGCGCACCAAGCACTTCATC
bmm	CTGAAGGGACCCAGGGAGTA	GTCTCCTCTGCGATTTGCCAT
Ilp2	ACGAGGTGCTGAGTATGGTGTGCG	CACTTCGCAGCGGTTCCGATATCG
Thor	AACCCTCTACTCCACCACTC	CAATCTTCAGCGACTTGG
Ilp6	TGCTAGTCCTGGCCACCTTGTTCG	GGAAATACATCGCCAAGGGCCACC

Supplementary Table 2: Primers pairs used for qPCR in this study.

shRNA name	*oligo forward*	*oligo reverse*
Bmm-shRNA-jz	*ctagcagtGTAATGGTGGAGGACTTAATTtagttatattcaagcataAATTAAGTCCTCCACCATTACgcg*	*aattcgcGTAATGGTGGAGGACTTAATTtatgcttgaatataactaAATTAAGTCCTCCACCATTACactg*

Supplementary Table 3: Sequence details for LexAop shRNA, the antisense sequence is AATTAAGTCCTCCACCATTAC.

**Name**	**Per liter**
deactivated yeast	18g
soy flour	10g
cornmeal	80g
malt	40g
corn syrup	5%
propionic acid	0.3%
nipagin	0.2%

Supplementary Table 4: Standard laboratory *Drosophila* food (per liter, 1% agar

**Name**	**Per liter**
$CaCl_2$ (2.5g per 100ml)	0.5 ml
$MgSO_4$ (25g per 100ml)	0.5 ml
$CuSO_4$ (0.25g per 100ml)	0.5 ml
$FeSO_4$ (2.5g per 100ml)	0.5 ml
$MnCl_2$ (0.1g per 100ml)	0.5 ml

$ZnSO_4$ (2.5g per 100ml)	0.5 ml
$MoNa_2O_4$ (20g per 100ml)	0.5 ml
Base buffer (10X)	100 ml

Supplementary Table 5: Protocol for modified starvation food (per liter)

**Base buffer (10x)**	**Per liter**
Acetic acid	30 ml
KH_2PO_4	30 g
NaHCO3	10 g

Supplementary Table 6: Protocol for 10X Base buffer (per liter)
